# Anti-Bacterial Properties and Hemocompatibility of Alkali Treated Nano-Structured Micro-Porous Titanium Surfaces

**DOI:** 10.3390/biomimetics10020115

**Published:** 2025-02-17

**Authors:** Aniruddha Vijay Savargaonkar, Emma Holloway, Liszt Y. C. Madruga, Bruno L Pereira, Paulo Soares, Ketul C. Popat

**Affiliations:** 1 Department of Mechanical Engineering, Colorado State University, Fort Collins, CO 80523, USA; aniruddha.savargaonkar@colostate.edu; 2 Department of Chemical and Biological Engineering, Colorado State University, Fort Collins, CO 80523, USA; 3 Department of Bioengineering, George Mason University, Fairfax, VA 22030, USA; 4 Department of Mechanical Engineering, Pontifícia Universidade Católica do Paraná, Curitiba 80215-901, PR, Brazil

**Keywords:** titanium, nanostructures, biomaterials, anti-bacterial studies, hemocompatibility

## Abstract

Titanium and its alloys have been the material of choice for orthopedic implants due to their excellent physical properties as well as biocompatibility. However, titanium is not able to integrate with bone due to the mismatch of mechanical properties. Additionally, bone has a micro–nano hierarchy, which is absent on titanium’s surface. A potential solution to the former is to make the surfaces porous to bring the mechanical properties closer to that of the bone, and a solution for the latter is to fabricate nanostructures. In this study, micro-porous titanium surfaces were hydrothermally treated using an alkali medium to fabricate nanostructures on the existing micro-porosity of the surface. The surface properties were evaluated using scanning electron microscopy, X-ray photoelectron spectroscopy, X-ray diffraction, and nanoindentation. The anti-bacterial properties of the surfaces were evaluated against Gram-positive and Gram-negative bacteria using fluorescence microscopy and scanning electron microscopy. Blood clotting is shown to improve the surface-to-bone integration; hence, whole blood clotting and platelet adhesion and activation were evaluated using a whole blood clotting assay, fluorescence microscopy, and scanning electron microscopy. The results indicate that nanostructured micro-porous titanium surfaces display significantly enhanced anti-bacterial properties as well as equivalent blood clotting characteristics compared to non-porous titanium surfaces.

## 1. Introduction

Titanium and its alloy, Ti-6Al-4V, have been extensively used as the material of choice for orthopedic implants due to their excellent physical properties such as good corrosion resistance, low modulus of elasticity, and considerable fatigue strength [1]. In addition to having desirable mechanical properties, titanium also has biocompatibility and is non-toxic [2,3]. Studies show that micro-roughness on implant surfaces leads to enhanced bone–implant contact and improved anchorage of the implant within the bone [1,4,5]. This micro-roughness can be achieved by various techniques like acid etching, grit blasting, plasma spraying, and anodization, among others [1]. The rough surfaces also increase the contact area between the implant and osteoblasts, which accelerates bone healing and leads to enhanced osseointegration. Osseointegration is defined as the direct structural and functional connection between the living bone and surface of a load-bearing implant [6], and is one of the key factors in determining the long-term success of the implant. To ensure a good connection, it is critical that the implant material resemblances the properties of the bone. Hence, there is a constant need to modify the material’s properties to facilitate better osseointegration. One of the major issues with using dense materials is the biomechanical mismatch of the elastic modulus, as well as the difference in the internal structure [7]. The inherent elastic modulus of dense Ti-6Al-4V is around 110 GPa [8], which is still about five times that of the maximum elastic modulus of bone.

A method to reduce the elastic modulus while preserving the other properties of titanium is changing the surface microporosity. This increases the surface area and may make the structure similar to that of the bone, as well as mimicking other mechanical properties of the bone tissue. A microporous structure is also favorable for cell growth and proliferation [9]. Microporous structures are better at osseointegration as compared to dense structures, as the interconnectivity allows for cell in-growth and improved migration, leading to improved integration of the implant with the bone [10]. In addition to reducing the elastic modulus and enhancing osseointegration, the addition of porosity also increases the micro-roughness of the surface. However, to achieve proper osseointegration, it is also important for the material’s surface to have the micro–nano hierarchy seen in natural bone structure [3]. Hence, there is a need to create nano-topography on microporous surfaces. Several different nano-level features have been fabricated and investigated, such as nanotubes, nanorods, nanowire, and nanoflowers [11,12,13,14]. Nano-level topographical surface features have displayed reductions in bacterial growth by disrupting the bacteria membrane, as well as displayed enhanced mesenchymal stem cell growth [15,16,17,18]. It is important for mammalian cells to adhere and proliferate before bacteria manage to adhere to prevent formation of biofilm on the surface [18]. Thus, it is important to develop surfaces that encourage cell adhesion and proliferation while preventing bacterial adhesion. Studies have also shown that surfaces can be modified by incorporating metal ions like silver and strontium to enhance their anti-bacterial properties and enhance bone growth, respectively [19,20]. Antibiotic drugs like gentamicin and ciprofloxacin have also been modified with porous titanium to lower bacterial adhesion [20,21].

Blood clotting on the surfaces also influences the osseointegration process of implants, as blood clotting is the first step in the wound healing process [22,23]. Blood clotting is caused mainly by the adhesion and activation of platelets on the surfaces. The rate of platelet adhesion and activation is influenced by surface topography and chemistry [24,25]. Removal of a blood clot has shown delayed bone growth in animal models [26]. As the blood clot is composed of biological matter, it serves as a source of growth factors and signaling molecules for the cells in addition to serving as a three-dimensional matrix for cell growth [24]. Platelets have also been shown to enhance the differentiation of mesenchymal stem cells to osteoblasts through facilitating the supply of growth factors in the form of platelet-rich plasma (PRP) [22]. Surface topography and chemistry are critical in influencing the blood clotting properties, with previous research showing that hydrophilic surfaces and surfaces with nano-topography or increased roughness enhance blood clot formation [27,28].

In this study, nanostructures were fabricated on microporous surfaces through hydrothermal treatment in an alkali medium to give them a micro–nano hierarchy. Hydrothermal treatment is a simple single-step technique which fabricates TiO_2_ nanostructures that have shown a decrease in bacterial adhesion while enhancing osteoblast response [29,30,31,32]. Nano-roughness has been shown to lower the bacteria adhesion to surfaces for *Staphylococcus aureus* and *Pseudomonas aeruginosa*, which are the most notorious bacteria and are responsible for majority of infections [16,33]. The anti-bacterial properties for the surfaces fabricated in this study were evaluated using Gram-positive bacteria and Gram-negative bacteria. Fluorescence microscopy was used to evaluate the growth of the bacteria, and scanning electron microscopy (SEM) was used to evaluate the biofilm formation on the surfaces. In addition to evaluating the anti-bacterial properties, the interaction between blood and its components on the surfaces was also investigated. Blood clotting was evaluated through whole blood clotting assays and platelet adhesion and activation. Fluorescence microscopy and SEM were used to evaluate the platelet adhesion and activation, respectively. The results show that microporous surfaces with nanostructures have significantly enhanced anti-bacterial properties compared to titanium with comparable blood clotting characteristics.

## 2. Materials and Methods

### 2.1. Fabrication of Nanostructured Micro-Porous Titanium Surfaces

Nanostructured micro-porous titanium (NPTi) surfaces were fabricated on commercially available micro-porous titanium (PTi) disks using hydrothermal treatment (HT). The PTi disks were cleaned in deionized (DI) water before subjecting them to HT with 25% NaOH solution. The HT was carried out in a hydrothermal autoclave reactor at 60 °C for 24 h [30]. After the HT, the surfaces were rinsed in DI water, followed by annealing at 530 °C for 3 h in an ambient oxygen environment. The temperature increment rate was 15 °C/min at the beginning of the annealing process.

### 2.2. Characterization of Nanostructured Micro-Porous Titanium Surfaces

The morphology of all the surfaces was characterized using JEOL 6500 field emission scanning electron microscopy (SEM). Before imaging, surfaces were gold-coated with a 10 nm layer using a sputter coater to enhance conductivity. The SEM parameters were optimized and chosen as follows: accelerating voltage of 15 kV, working distance of 10 mm, and vacuum pressure below 3 × 10^−4^ Pa. The images were collected at 100× and 5000× magnification.

The elemental composition of different surfaces was characterized using a PHI-500 X-ray photoelectron spectroscopy (XPS) probe which was equipped with Al Kα X-ray source. Survey spectra were collected from 0 to 1100 eV at a pass energy of 187 eV. The data were analyzed, and the elemental percentage present on surfaces was calculated using CASA XPS software 2.3.24PR1.0.

The surface crystallinity was characterized using thin-film X-ray Diffraction, where the equipment used was XRD-7000 from Shimadzu. Spectra were collected using CuKα radiation (λ = 1.5406 Å) with continuous scanning at a scanning speed of 1°/min over a 2θ range from 20° to 80° at an incidence angle of 5°.

The surface mechanical properties of hardness and elasticity were characterized using nanoindentation. A Nanoindenter (Zwick-Roell/Asmec) was programmed with a 50 μm distance between each indentation with a 0.1 N maximum applied force on the surface using a calibrated Berkovich tip. A quasi-continuous stiffness measurement was used for indentation to allow for high accuracy. This is possible due to the progressive increase in force from 0 to 100 mN, along with a dwell time at each force point.

### 2.3. Bacteria Culture on Nanostructured Micro-Porous Titanium Surfaces

Investigating the anti-bacterial properties of different surfaces was performed by culturing two different strains of bacteria: *Staphylococcus aureus* (Gram-positive) and *Pseudomonas aeruginosa* (Gram-negative). Bacteria were incubated at 37 °C for 12 h in 8 mL solution of tryptic soy broth (TSB) to grow. The bacteria was allowed to grow till an optical density reading of 0.52 was achieved at a wavelength of 562 nm, equating it to a concentration of 10^9^ Colony Forming Unit (CFU)/mL of TSB solution. The solution was then diluted to 10^6^ CFU/mL of TSB to seed on the surfaces to evaluate the bacteria adhesion and growth. Prior to the bacteria seeding, surfaces were sterilized under UV light for 60 min and cleaned with DI water. Surfaces were then placed in a 24-well plate and a bacterial solution of 10^6^ CFU/mL was added in each well and incubated for 6 h and 24 h.

Fluorescence microscopy was used to characterize bacteria adhesion on different surfaces. Following the incubation period, the bacterial solution was removed, and surfaces were rinsed with PBS to remove any unadhered bacteria. The surfaces were stained for 15 min in a dark environment using a stain solution consisting of 3 μL/mL of propidium iodide and Syto9 stains with a 1:1 ratio in PBS. After the incubation, the stain was removed and the surfaces were washed and stored in PBS. The surfaces were imaged using a fluorescence microscope (Zeiss Axiovision) at 20× magnification.

SEM was used to characterize bacteria morphology and biofilm formation on surfaces. Following the incubation period, the bacterial solution was removed, and surfaces were rinsed with PBS. Bacteria on surfaces were fixed by immersing them in a fixative solution comprising 3% glutaraldehyde, 0.1 M sucrose, and 0.1 M sodium cacodylate in DI water for 45 min. Following the fixation, the surfaces were immersed in a buffer solution (fixative solution without glutaraldehyde) for 10 min. Subsequently, the surfaces were immersed in ethanol solutions of increasing concentration from 35%, 50%, 70%, and 100% for 10 min each. The surfaces were stored in a desiccator before taking SEM images. The surfaces were gold-coated with a 10 nm layer before SEM imaging to increase the conductivity. SEM images were captured at 2500× and 10,000× magnification. The SEM parameters were chosen as follows: accelerating voltage of 15 kV, working distance of 10 mm, and vacuum pressure below 3 × 10^−4^ Pa [16].

### 2.4. Platelet Adhesion and Activation on Nanostructured Micro-Porous Titanium Surfaces

Whole human blood was drawn by a trained phlebotomist with formal consent from healthy individuals. The protocol used was approved by the Institutional Review Board at Colorado State University, which follows the National Institutes of Health’s ‘Guiding Principles for Ethical Research’. To avoid clotting, the whole blood was drawn into 10 mL vacuum tubes coated with ethylenediaminetetraacetic acid (EDTA). PRP was obtained by centrifuging the EDTA-coated tubes for 15 min at 150 g. The surfaces were incubated for 2 h at 37 °C and 5% CO_2_ in a 48-well plate.

Fluorescence microscopy was used to characterize platelet adhesion. After the incubation period, surfaces were rinsed twice with PBS to wash the unadhered platelets, and platelets were stained with 5 μM solution of calcein for 20 min at room temperature in a dark environment. After the staining, surfaces were washed and stored in PBS before being imaged. ImageJ was used to quantify the platelet adhesion on the surfaces. SEM was used to characterize platelet activation. Following the incubation, the surfaces were rinsed with PBS and fixed using a similar process as described earlier. The surfaces were coated with gold and imaged in SEM with parameters as described earlier at magnifications of 500× and 5000×.

### 2.5. Whole Blood Clotting on Nanostructured Micro-Porous Titanium Surfaces

Whole blood was collected in uncoated 3 mL vacuum tubes. Whole blood clotting was evaluated by placing 7 μL of blood on different surfaces in a 24-well plate. The blood was allowed to clot for 15, 30, and 45 min. After the time interval, 700 μL DI water was added gently to the wells, and surfaces were gently agitated for 5 min on a shaker to lyse the red blood cells and release hemoglobin. Blood which was not exposed to any surface was used as control (100% hemoglobin release). The absorbance of hemoglobin was measured using a plate reader at 540 nm. The control was read immediately after collection at 0 min [34,35].

### 2.6. Statistical Analysis

Surface characterization was repeated on at least two different samples of each surface. SEM imaging and XPS data were collected at two different locations on each sample, and this was repeated for three samples of each surface (n_min_ = 6). XRD data were collected at two different locations on each sample, and this was repeated for two samples of each surface (n_min_ = 4). Bacteria studies were performed two times with at least three different samples of each surface (n_min_ = 6). Blood studies were performed two times with at least three different samples of each surface (n_min_ = 6). The quantitative results were analyzed using two-way analysis of variance (ANOVA) and Tukey’s honestly significant difference (HSD) test using the JMP software, with significant results considered when *p* < 0.05. The data presented here are from one study, and similar trends were observed from other bacteria studies as well.

## 3. Results and Discussion

**Figure 1:**
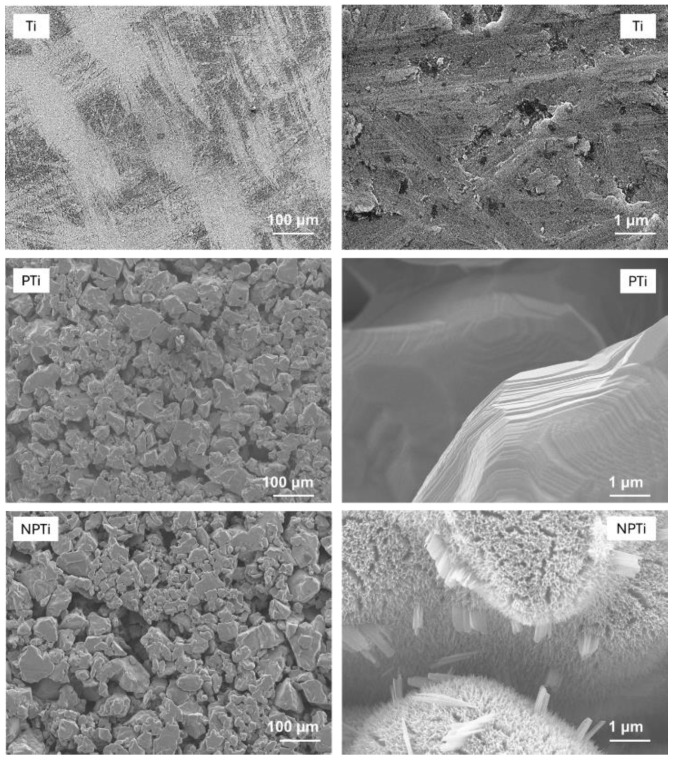
Representative SEM images of different surfaces at 100× and 5000× magnification.

Microporous titanium surfaces were hydrothermally treated in an alkali (NaOH) medium to fabricate nano-structured topography on the micro-porous surface to mimic the micro–nano hierarchy of the bone. HT etches the surface to form nanostructures on the surface [36]. Surface topography is critical in determining the interaction of cells and proteins with the surface. SEM was used to characterize the surface topography of the titanium and microporous surfaces before and after HT (Figure 1). As expected, Ti surfaces did not have any distinct features, and the roughness can be attributed to the cleaning process for the Ti. The HT resulted in uniform surfaces that did not change the inherent micro-porosity of the surface (NPTi) when compared to unmodified surfaces (PTi), as evident from low-magnification SEM images. However, at higher-magnification, nanostructures are visible on NPTi. There is “web”-like structure on the surface composed of oxide. The presence of “petal”-like features on the NPTi can also be attributed due to dissolution and precipitation mechanism at lower concentrations of alkali solution [29]. For this study, 25% NaOH solution in DI water (*v*/*v*) was used, and the HT was carried out at 60 °C for 24 h. These parameters were chosen after several iterations with the goal of fabricating the nano-topography without compromising the existing micro-porosity of the surface. Other studies which performed this alkali-based HT have also reported a similar web-like structure formation [30,37,38,39].

**Figure 2:**
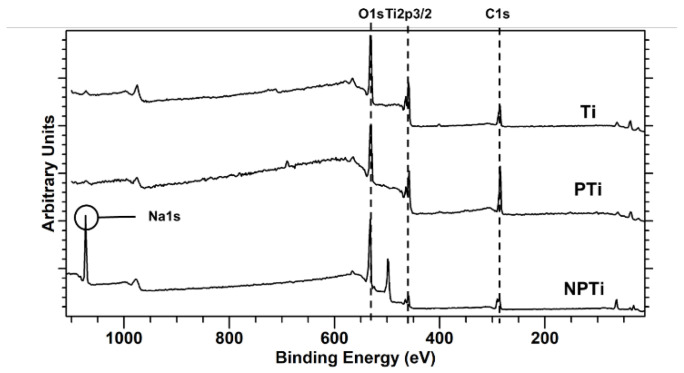
XPS Spectra for different surfaces.

Surface chemistry plays a critical role in determining the biological response from the cells, and hence it was analyzed. XPS was used to characterize the surface chemistry of different surfaces (Figure 2). The survey spectra were collected, and analysis was carried out using the CASA XPS software (Table 1). As expected, all surfaces displayed the presence of Titanium (Ti2p3/2), Carbon (C1s), and Oxygen (O1s). The presence of carbon is due to the possible carbon contamination in the XPS chamber and surface impurities. The carbon content on PTi surfaces is lower compared to Ti due to its porous structure. NPTi surfaces have the lowest carbon content, which can attributed to the HT. NPTi surfaces also displayed the presence of sodium (Na1s) due to HT. The titanium on the microporous surface reacts with the NaOH solution, which leads to the formation of sodium titanate on the surface. This reaction is dependent the NaOH concentration, temperature, and the time of the HT [31,36].

**Figure 3:**
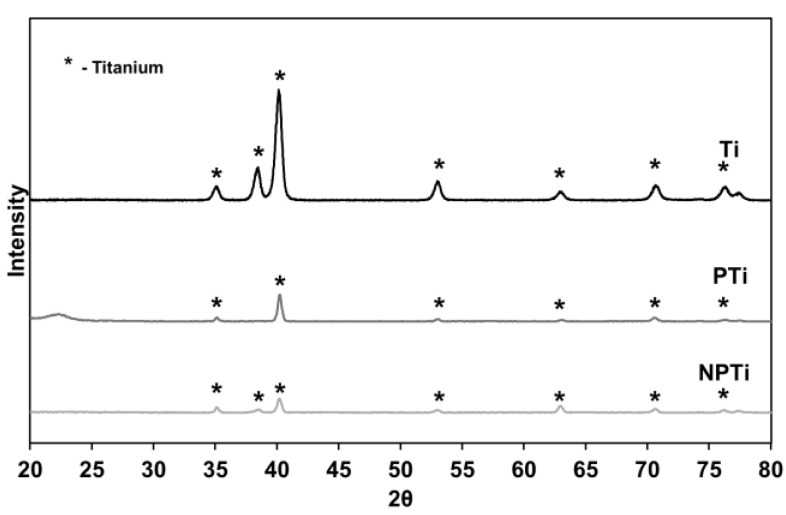
XRD Spectra for different surfaces.

Surface crystallinity influences surface properties, such as hardness, diffusion, and density, that, in turn, affects cell and protein interactions with surfaces [40]. Furthermore, HT may also affect the crystal lattice structure. Thus, XRD was used to evaluate surface crystallinity (Figure 3). As expected, all of the surfaces displayed titanium peaks. The lower peak intensity of PTi and NPTi can be attributed to higher scattering occurring for these surfaces as compared to Ti surfaces. Hence, as higher scattering of X-ray occurs, a lower number of x-rays reach the detector. NPTi surfaces also did not experience a change in the titanium phase; this has also been observed in other studies, where a similar HT was carried out [41]. This proved that the HT only affected the surface and not the crystal lattice.

A strong argument for using porous surfaces for implants is the mismatch between the elastic modulus of the bone and titanium and its alloy, like Ti-6Al-4V. This mismatch leads to issues like aseptic loosening and even implant failure [42]. A lower elastic modulus has also demonstrated enhanced anti-bacterial properties in previous studies [43,44]. The nanoindentation technique was used to calculate the hardness of the surface (H) and elastic modulus (E) for all of the surfaces (Table 2). As expected, the E values for PTi and NPTi surfaces are lower than Ti surfaces. E values for NPTi are lower as compared to PTi, which can be attributed to the HT and fabrication of nanostructures. The H and E values for bone were obtained from previous studies for comparison. The combination of microporosity and nanostructures lowers the H and E value closer to the bone compared to Ti surfaces. However, it still is almost three times that of the bone.

**Figure 4:**
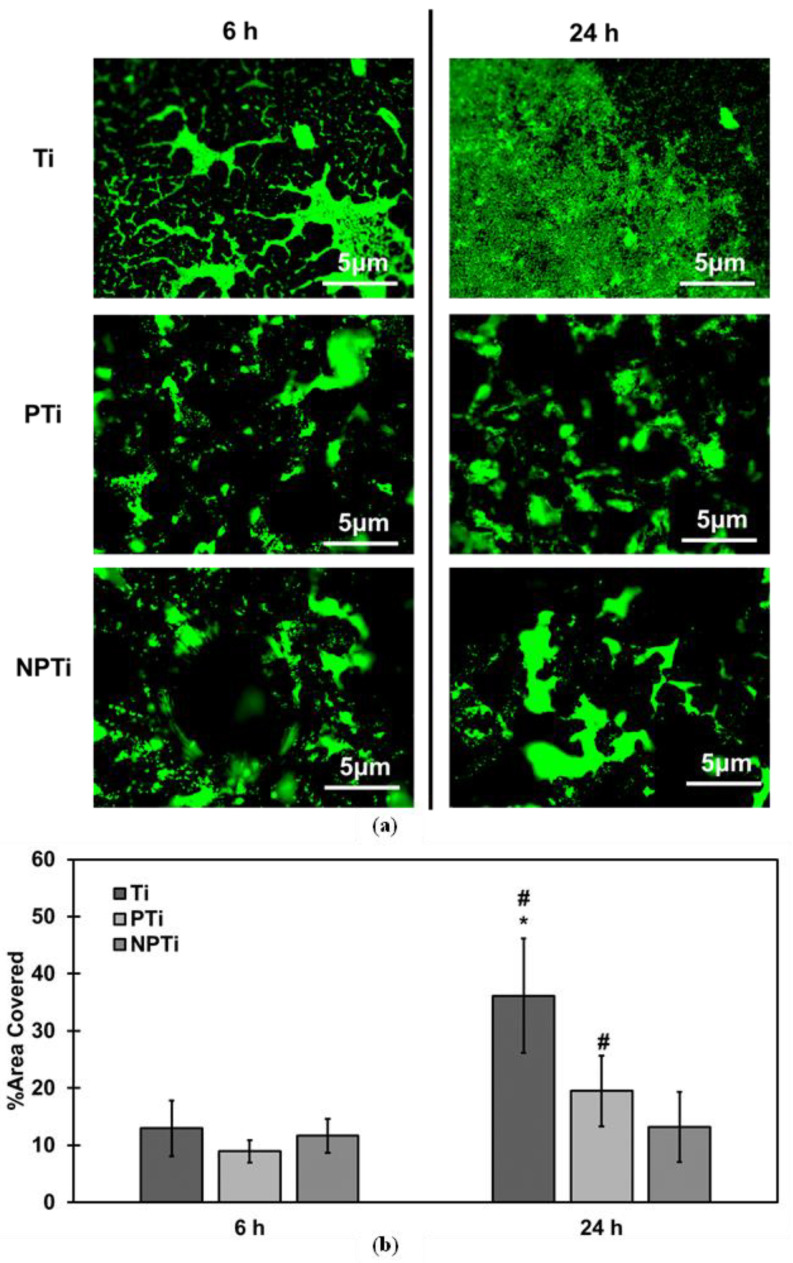
(**a**) Representative fluorescence microscopy images at 20× magnification for *Staphylococcus aureus* adhesion and growth on different surfaces. (**b**) Quantification of fluorescence microscopy images using ImageJ software indicating live bacteria adhesion on different surfaces [* & # denotes *p* < 0.05].

Anti-bacterial properties are critical for the long-term success of the implant, and hence were investigated using *Staphylococcus aureus* (Gram-positive bacteria) and *Pseudomonas aeruginosa* (Gram-negative bacteria). *Staphylococcus aureus* is the most antibiotic-resistant bacteria, while *Pseudomonas aeruginosa* forms robust biofilms and has the ability to resist antibiotics [47,48]. Bacteria adhesion and growth was evaluated using fluorescence microscopy. Syto9 stain was used, which penetrates the cell wall and stains live cells green [49]. For *Staphylococcus aureus*, the fluorescence microscopy results indicate an increase in bacteria adhered from 6 h to 24 h of culture (Figure 4a). Ti surfaces have the highest bacteria growth among all of the surfaces, whereas PTi and NPTi surfaces had marginal bacteria growth from 6 h to 24 h of culture. After 6 h of culture, all of the surfaces had similar adhesion and growth of bacteria. However, after 24 h of culture, Ti surfaces displayed increased growth as compared to both PTi and NPTi surfaces. The fluorescence images were analyzed using ImageJ software to quantify bacteria adhesion (Figure 4b). After 6 h of culture, all of the surfaces had similar percentages of bacteria adhesion. However, from 6 h to 24 h of culture, there was drastic bacteria growth. Ti surfaces experienced the highest bacteria growth, almost three times growth from 12% to 36%, followed by PTi surfaces, which experienced almost double growth from 8% to 19%. NPTi surfaces, however, experienced lowest growth of 2% from 11% to 13%. There is also a significant difference between bacteria growth on non-porous and porous surfaces (*p* < 0.05). The difference in bacteria adhesion between non-porous and porous is due to the presence of porosity, which has also been demonstrated in other studies [10]. The surface topography of NPTi surfaces does not allow for the bacteria to adhere to the surface, hence leading to lower adhesion and, therefore, growth. This decreased bacteria growth on the NPTi surfaces compared to PTi surfaces has also been demonstrated in previous studies performed on titanium using HT in an alkali medium [32].

**Figure 5:**
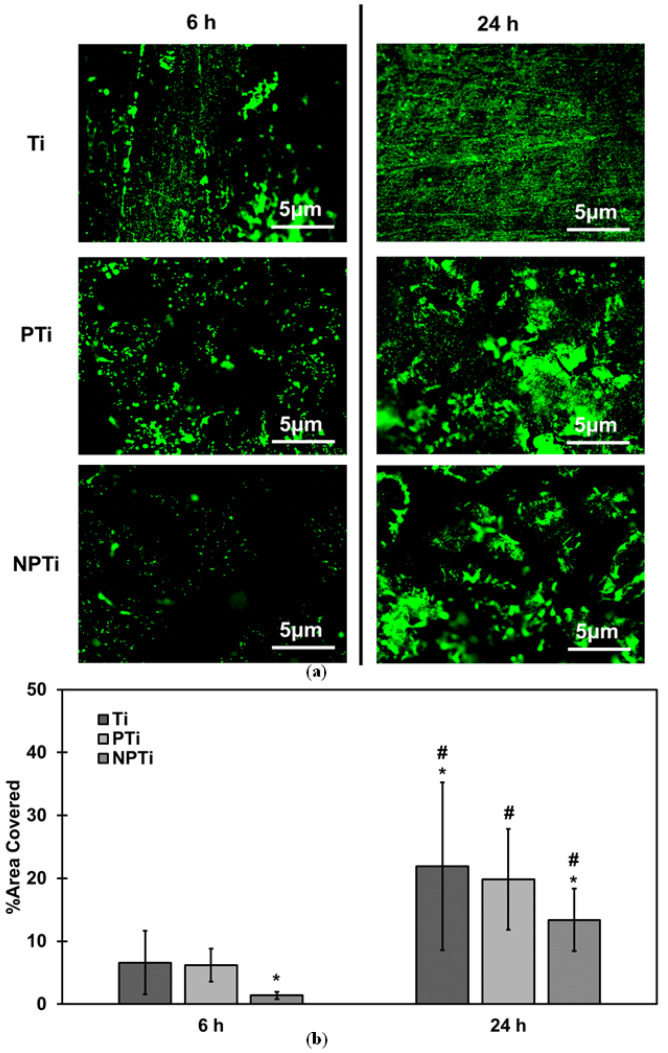
(**a**) Representative fluorescence microscopy images at 20× magnification for *Pseudomonas aeruginosa* adhesion and growth on different surfaces. (**b**) Quantification of fluorescence microscopy images using ImageJ software, indicating live bacteria adhesion on different surfaces [* & # denotes *p* < 0.05].

The fluorescence microscopy results for *Pseudomonas aeruginosa* were similar to that of *Staphylococcus aureus* (Figure 5a). After 6 h of culture, Ti and PTi surfaces had similar bacteria adhesion compared to the NPTi surfaces where the bacteria adhesion was less. However, after 24 h of culture, there was enhanced growth on all the surfaces, with Ti demonstrating the most growth followed PTi and NPTi surfaces, respectively. The quantified data from the fluorescence microscopy (Figure 5b) show that there is significantly less adhesion on NPTi surfaces as compared to Ti and PTi surfaces after 6 h of culture (*p* < 0.05). After 24 h of culture, as expected, Ti surfaces experienced the highest growth by almost four times from 6% to 22%, followed by PTi, which experienced three times growth from 6% to 19%, and with NPTi experiencing the most drastic growth from 2% to 13%. These data are similar to the one observed for *Staphylococcus aureus*. As demonstrated in other studies which have fabricated and tested similar surfaces [32], NPTi surfaces displayed a higher anti-bacterial activity against *Pseudomonas aeruginosa*. This has been attributed to *Pseudomonas aeruginosa* being a gram-negative bacteria with a thinner cell wall, hence making it more susceptible to the nanostructures [50]. Hence, this shows that NPTi surfaces demonstrate significantly increased anti-bacterial properties against gram-positive as well as gram-negative bacteria.

**Figure 6:**
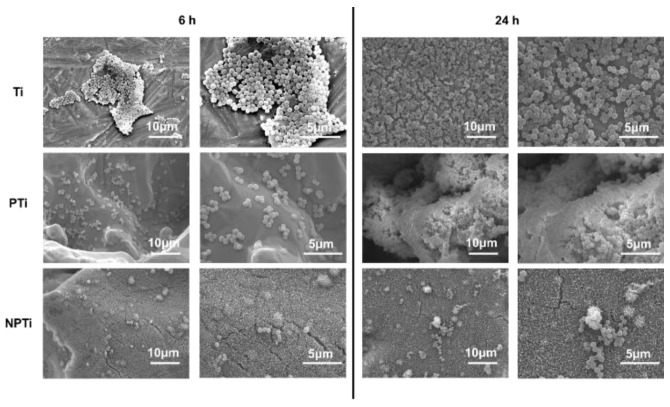
Representative SEM images for *Staphylococcus aureus* on different surfaces at 2500× and 10,000× magnifications.

Biofilm is a natural polymer which is secreted after bacteria have attached and proliferated on a surface. For the long-term success of implants, it is important to prevent biofilm formation. In this study, the biofilm formation on different surfaces was characterized using SEM. The SEM images for *Staphylococcus aureus* are in agreement with the fluorescence microscopy images (Figure 6). After 6 h of culture, Ti surfaces had the most bacteria adhesion, with clusters forming on the surface. PTi surfaces had similar cluster formation; however, they were smaller, with less bacteria adhering to the surface. NPTi surfaces had the lowest adhesion of the bacteria cells, with almost no clusters forming and with mainly isolated bacteria attached to the surface. However, after 24 h of culture, a drastic growth of bacteria is visible on the Ti and PTi surfaces. Bacteria completely covered the surfaces, and there was biofilm formation observed on the Ti surfaces, with additional bacteria clusters forming on the film. PTi surfaces were also covered with bacteria, despite their micro-porous topography. In contrast, NPTi surfaces have less bacteria growth, with smaller clusters forming on the surfaces without any biofilm formation.

**Figure 7:**
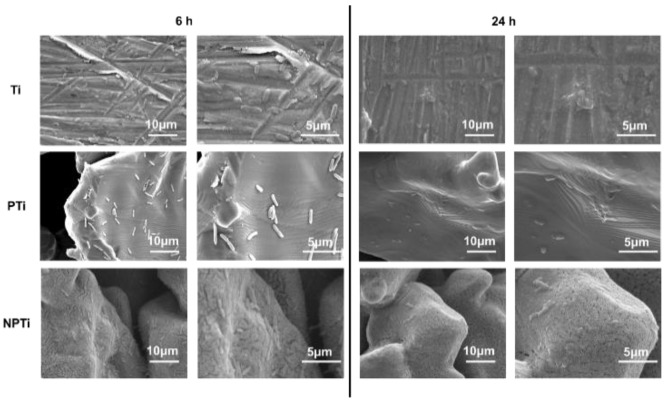
Representative SEM images for *Pseudomonas aeruginosa* on different surfaces at 2500× and 10,000× magnifications.

The SEM images for *Pseudomonas aeruginosa* were also in agreement with the data obtained from fluorescence microscopy (Figure 7). After 6 h of culture, Ti and PTi surfaces show an equivalent coverage of bacteria. Ti surfaces also displayed some biofilm formation, which can be seen in the higher-magnification images. NPTi surfaces, on the other hand, had a lower bacteria attached to them. PTi as well as NPTi surfaces did not show any biofilm formation. After 24 h of culture, all of the surfaces displayed an enhanced bacterial growth. Ti and PTi surfaces were completely covered with biofilm. NPTi surfaces, however, did not have any biofilm formation on them. On the contrary, the bacteria which had adhered to the NPTi surfaces displayed membrane deformation. As mentioned earlier, there was an improved activity observed against Gram-negative bacteria on NPTi surfaces due to their thinner membrane.

**Figure 8:**
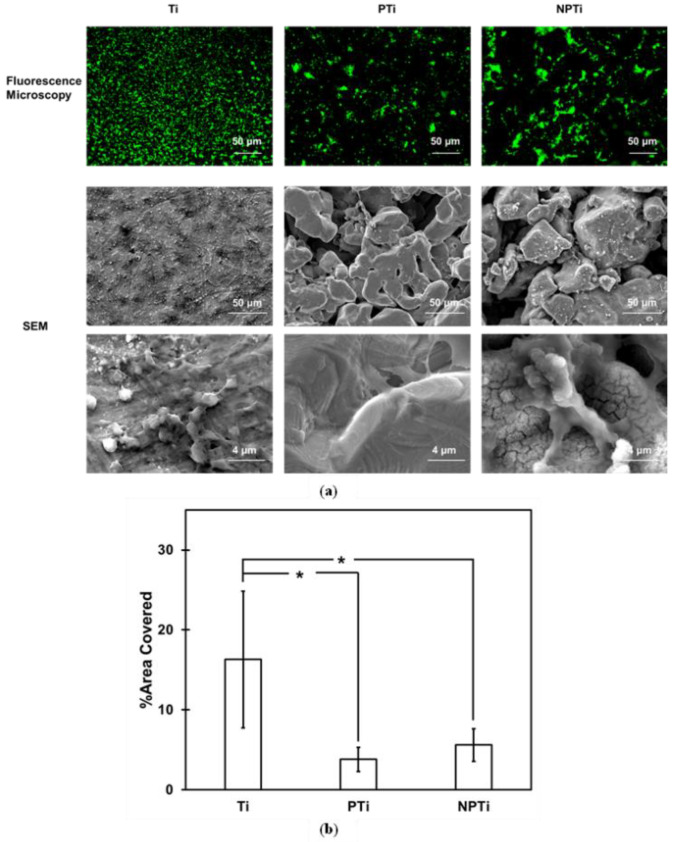
(**a**) Representative fluorescence microscopy images at 10× magnification and SEM images at 500× and 5000× magnification for platelet adhesion and activation. (**b**) Platelet adhesion on different surfaces [* denotes *p* < 0.05].

Blood clotting on implant surfaces has demonstrated enhanced osteoconduction, which paves the way for the increased recruitment and migration of osteogenic cells [24]. Formation of blood clots allows for the migration of cells which adhere on the surface, leading to mineralization and, consequently, bone formation [22]. PRP has demonstrated faster differentiation of mesenchymal stem cells and providing growth factors, hence making it critical to evaluate their behavior [22]. Platelet adhesion and activation on different surfaces were characterized using fluorescence microscopy and SEM, respectively (Figure 8a).

All of the surfaces had platelet adhesion on them (green), with Ti surfaces having the most coverage, followed by NPTi surfaces and then PTi surfaces. The fluorescence microscopy data were quantified using ImageJ software (Figure 8b). Ti surfaces had significantly increased adhesion of platelets, being 16%, compared to PTi and NPTi surfaces at 4% and 6% (*p* < 0.05). NPTi surfaces had, however, higher platelet adhesion compared to PTi surfaces, which can attributed to the HT, and has been observed in other studies as well [51]. Clearly formed clots were visible on the NPTi surfaces with adhered and activated surfaces, which has been attributed to the changes in surface chemistry brought about due to the HT. Platelet activation was characterized using SEM. Surfaces displayed clear morphological differences in the deposits formed due to the activation of platelets. PTi and NPTi surfaces showed spreading when observed at high magnification, which was not visible on the Ti surfaces. At lower magnifications, the activated platelets observed were also in the similar order, as seen for the fluorescence microscopy images.

**Figure 9:**
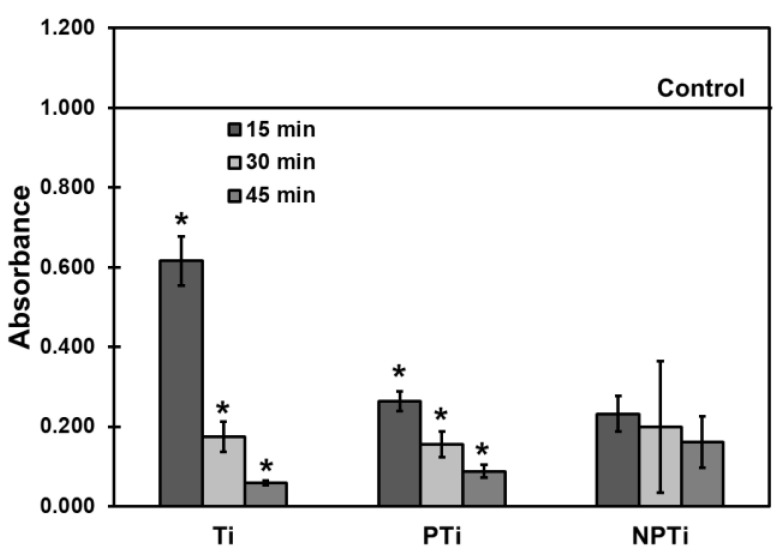
Whole blood clotting absorbance measurements for different surfaces [* denotes *p* < 0.05].

Investigating whole blood clotting is critical in accurately understanding its interaction with biomaterial. It is also more clinically relevant compared to platelet adhesion and activation, as it involves all of the components of blood [34]. Whole blood clotting was evaluated using an assay where free hemoglobin absorbance was measured after allowing for the blood to clot on surfaces for different time intervals (Figure 9). The amount of free hemoglobin released is inversely proportional to the clotting. If more blood clots, less hemoglobin will be released and, hence, absorbance values will be less. After 15 min, PTi and NPTi surfaces had a higher blood clotting compared to Ti surfaces. However, as more time was allocated for blood clotting, there was a significant difference in the amount of free hemoglobin observed for Ti surfaces from 15 min to 45 min. Similar behavior was also observed on PTi and NPTi surfaces, but it was not as profound, as seen on Ti surfaces. NPTi surfaces allowed for the blood to clot faster in the short term; however, with more time allowed for clotting, Ti and PTi surfaces demonstrated higher clotting. NPTi surfaces displayed a higher release of free hemoglobin compared to PTi surfaces. This has also been observed in other studies where alkali-based HT was performed on the surfaces [37].

## 4. Conclusions

Porous titanium has been proposed as a solution to counter the mismatch of the mechanical properties between the implant and bone which makes the process of integration with bone challenging. Hence, in this study, microporous titanium surfaces were modified using alkali hydrothermal treatment to fabricate nanostructures. The purpose of nanostructures is two-fold: mimicking bone structure, and implementing an additional layer of difficulty for the bacteria to adhere. The SEM results display no change in surface topography at lower magnification, indicating that the HT did not affect the microporosity of the surfaces. XPS results show the presence of titanium on all the surfaces and sodium on NPTi surfaces due to the fabrication of sodium titanate due to HT. XRD was performed to understand if the HT modified the crystallinity of the surfaces. There was no change in the crystallinity, with all the surfaces showing titanium peaks. The effect of microporosity and HT on the mechanical properties was also evaluated. The results demonstrate a decrease in the mechanical properties from Ti > PTi > NPTi, showing that the porosity and HT brought the surfaces closer to the bone in terms of mechanical properties compared to Ti surfaces, even if there was some mismatch. The anti-bacterial properties of the surfaces were evaluated against *Staphylococcus aureus* (Gram-positive bacteria) and *Pseudomonas aeruginosa* (Gram-negative bacteria). After 6 h and 24 h of culture, the results show that NPTi surfaces significantly performed better in comparison to other surfaces. The blood clotting properties of the surfaces were evaluated through platelet adhesion and activation and a whole blood clotting assay. The results show that NPTi surfaces had equivalent blood clotting compared to Ti surfaces. This study demonstrates that NPTi surfaces have the potential to be surfaces for orthopedic implants.

## Figures and Tables

**Table 1:** Surface elemental composition for different surfaces.

	% Ti2P3/2	% C1s	% O1s	% Na1s
Ti	7.38	57.43	35.20	-
PTi	14.15	38.56	47.30	-
NPTi	3.62	27.53	44.78	24.07

**Table 2:** Nanoindentation hardness (H) and elastic modulus (E) values for different surfaces.

Surface	Nanoindentation Hardness (GPa)	Elastic Modulus (GPa)
Ti	2.57 ± 0.54	104.85 ± 14.56
PTi	3.38 ± 1.63	79.66 ± 33.50
NPTi	1.18 ± 0.16	62.27 ± 8.05
Bone	0.43 + 0.06 [45]	21.2 + 5.3 [46]

## Data Availability

The datasets used and analyzed during the current study are available from the corresponding authors upon reasonable request.
